# The Effect of *PRKAA2* Variation on Type 2 Diabetes Mellitus in the Asian Population: A Systematic Review and Meta-Analysis

**DOI:** 10.21315/mjms2022.29.3.2

**Published:** 2022-06-28

**Authors:** Dita Maria Virginia, Iwan Dwiprahasto, Mae Sri Hartati Wahyuningsih, Dwi Aris Agung Nugrahaningsih

**Affiliations:** 1 Faculty of Medicine, Public Health and Nursing, Universitas Gadjah Mada, Yogyakarta, Indonesia; 2 Faculty of Pharmacy, Sanata Dharma University, Yogyakarta, Indonesia

**Keywords:** AMP-activated protein kinase, genetic variation, type 2 diabetes mellitus, risk factor, Asian

## Abstract

The prevalence of type 2 diabetes mellitus (T2DM) is increasing among Asians. The adenosine monophosphate-activated protein kinase (AMPK) increases T2DM risk through insulin resistance. Glucose levels are related to AMPK subunit α2 encoded by *PRKAA2*. This systematic review and meta-analysis aimed to analyse the association between *PRKAA2* variation and T2DM risk. Publication search related to *PRKAA2* and T2DM used PubMed, ProQuest, and ScienceDirect databases. Article selection based on inclusion and exclusion criteria only included Japanese and Chinese populations. This meta-analysis used five genotype models to estimate the effect of *PRKAA2* variation and T2DM risk. Additionally, a fixed-effect model was selected to measure the pooled size effect if *P* > 0.05 or I^2^ < 50%. Qualitative analysis included four eligible studies, and meta-analysis included only two studies because both showed data concerning rs2746342 variation. Patients with G allele are 1.45 times more likely to have T2DM than patients with T allele (95% confidence interval [CI]: 1.20, 1.76; *P*: 0.0001). Notably, patients who had GG genotype have 1.96 times higher risk of T2DM compared with those with TT genotype (95% CI: 1.34, 2.87; *P*: 0.0005), dominant model (odds ratio [OR]: 1.75; 95% CI: 1.32, 2.31; *P*: 0.001), and recessive model (OR: 1.43; 95% CI: 1.01, 2.01; *P*: 0.04). *PRKAA2* variation, especially in rs2746342, has an association with T2DM risk in the G allele, additive, dominant, and recessive models. G allele might be the most contributable factor in increasing T2DM susceptibility.

## Introduction

Type 2 diabetes mellitus (T2DM) prevalence has increased almost fourfold from 1980 to 2014, especially in developing countries. The International Diabetes Federation declared that 463 million adults are diagnosed with T2DM, and it will reach 700 million by 2045. Globally, T2DM is one of the factors that increase the cardiovascular risks and cause morbidity and mortality. Approximately 50% of all deaths attributable to T2DM occur before 70 years old (1–2). Prevalence in Asia in 2017 was approximately 8.5% among patients aged 20 years old–79 years old (3). T2DM is a pathological metabolic condition indicated by hyperglycaemia, using A1c and fasting plasma glucose (FPG) as biomarkers (4). Several studies in Asia showed uncontrolled A1c and FPG lead to other complications (5–6). Early detection of T2DM continues to be a critical point of successful treatment. Genetic studies may present a path to discover a novel method for early detection (7).

T2DM is caused by several risk factors, such as environmental factors (including diet, physical activities and microbiome) and genetic factors (8). Environmental risk factors are recognised as contributors to T2DM development, including obesity, sedentary lifestyle and stress. These environmental factors obviously have a significant role in T2DM, but each person might have a different outcome. Even someone with similar environmental exposures might be more prone to develop T2DM than others and this increased risk seems to be inherited (9). The heritability of T2DM ranges from 26%–69% with age at onset (10–11). Accordingly, it is important to have an investigation about genetics in patients with T2DM. Genetic variation, such as single-nucleotide polymorphism (SNP), could be a genetic marker for early detection of diseases (12), including T2DM, so it could assist in predicting T2DM risk. It is promising to develop a method to detect T2DM risk based on genetic variation models, which could provide early prevention to delay the onset of T2DM among nondiabetic persons who have a risk genetically.

The Genome-Wide Association study has examined several genes that contribute to the development of T2DM. Genes related to the pathophysiology of T2DM, including insulin secretion whether in β cell or gluconeogenesis, insulin sensitivity in muscles, fat synthesis and distribution have become the targets of pharmacogenetics study (13). The genetic variation might disturb the function or binding sites of those proteins that contribute in T2DM pathogenesis (14). Some genes such as *KCNQ1*, *TCF7L2*, *KCNJ11*, *IRS1*, *MTNR1B* and *PPARG2* have been already observed related to T2DM risk (15).

There are various genetic determinants involved in the pathogenesis of T2DM. The adenosine monophosphate-activated protein kinase (AMPK) is one of the most important proteins that is widely considered to be related to T2DM. Some reviews and research showed an association between AMPK and T2DM risk through insulin resistance pathways (16–17).

AMPK is a heterotrimeric protein complex that consists of catalytic subunit α (α1 and α2), scaffolding subunit β (β1 and β2) and regulatory subunit γ (γ1, γ2, and γ3). AMPK might be related to T2DM through suppression of gluconeogenesis in the liver, glucose uptake in muscles and lipid metabolism (18–20). AMPK activation is known through allosteric changes in the AMPK complex, which increases phosphorylation of the α subunit in Thr-172, either through upstream kinase phosphorylation or preventing dephosphorylation through protein phosphatase (21–22). AMPK phosphorylation occurs in the α subunit (23), which has been shown to be more involved in reducing glucose and insulin resistance compared with α1 (24). Research results indicate the *PRKAA2* encoding AMPKα2 is possibly related to T2DM pathogenesis.

*PRKAA2* is located at chromosome 1p31 in regions 56645317 to 56715335 and consists of 12 exons. Although several genetic variations are located on the non-coding region of the *PRKAA2*, several studies detected that the genetic variation in the intron is correlated with T2DM. Most of the studies concerned about *PRKAA2* variations focused on the positive association with T2DM. However, another study showed a negative insignificant association. Some factors, such as ethnicity or sample size, might have caused those conflicting results. The purpose of this review and meta-analysis was to clarify the impact of *PRKAA2* variation on the risk of T2DM, especially in the Asian population. SNP rs2746342 is one of the most common genetic variations that was studied in Asians. Furthermore, the results of our systematic review of studies conducted on rs2746342 were further investigated in the meta-analysis. The conclusion support that the impact of *PRKAA2* variation could be a pathway to enable creating a model or tool to predict T2DM risk based on genetics.

## Methods

### Data Sources and Selection of Eligible Study

This study conducted a comprehensive literature search in PubMed, ProQuest and ScienceDirect databases to identify all relevant case-control studies. We included all publications before 24 January 2020. The following search terms were used: (*PRKAA2*) AND (polymorphism or SNP or genetic variation or rs or allele) AND (diabetes or diabetes mellitus or diabetic or type 2 diabetes mellitus or T2DM) AND (Asia or Asian). The title and abstract were skimmed for related content to confirm their relevance; then, potential articles were retained for further appraisal.

### Inclusion and Exclusion Criteria

All the eligible studies conformed to the following criteria: i) assess the association between *PRKAA2* variation and T2DM susceptibility; ii) case–control studies; iii) research on human patients; iv) determine the frequency of genotypes of all cases and controls; v) papers with the English language; and vi) full-text publications were included in this review. However, studies were excluded if meeting the following criteria: i) animal studies; ii) insufficient data of genotype frequency; and iii) focused on another disorder, instead of T2DM.

### Data Extraction

All authors independently assessed the articles and all unclear decisions finally obtained an agreement after discussion among all authors. This study extracted the following information from all included publications: name of the first author, year of publication, ethnicity, sample size, a method used to detect genetic variation, SNP, number of participants in cases (T2DM) and controls (non-T2DM), demographic characteristics (including gender, age and body mass index [BMI]), and cardiometabolic profiles (including FPG, total cholesterol [TC], triglycerides, high-density lipoprotein [HDL] and low-density lipoprotein [LDL]-c).

### Statistical Methods

SNP genotyping in the Hardy-Weinberg equilibrium (HWE) was performed using the Pearson’s Chi-squared test to determine genotype stability correlated with *PRKAA2* variation in the control group (significance level of HWE at *P* < 0.05). This review investigated the association between SNP *PRKAA2* and T2DM through pooled odds ratio (OR) and 95% confidence interval (CI) in all models (allele contrast, additive, dominant and recessive model). The significance of pooled OR was determined using the *Z* test (*P* < 0.05 considered statistically significant). The heterogeneity test was measured using I^2^ and showed significant heterogeneous results (*P* < 0.05 or I^2^ > 50%). We used a fixed-effect model if I^2^ < 50%, whereas I^2^ 50%–90% used a random-effects model. Different genetic models were applied to compare alleles and genotypes. This study conducted analysis with the allele contrast (G versus T allele), additive, dominant and recessive models. Subgroup analysis was conducted by sex with different genetic models, as mentioned above. This meta-analysis was conducted using Review Manager Software version 5.3 (RevMan v5.3, The Cochrane Collaboration, Oxford, UK) (25).

## Results

### Characteristics of Included Studies

**Figure 1:**
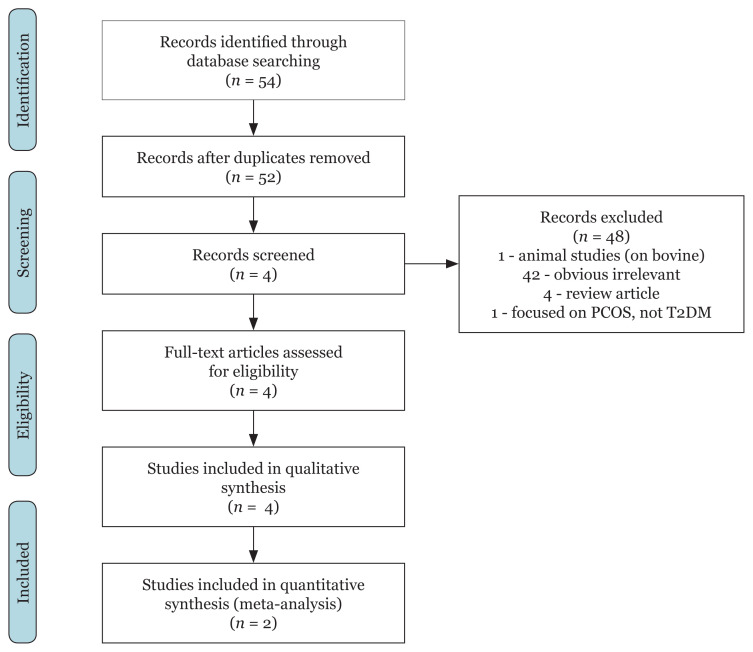
Flow chart of the literature selection process

The results of comprehensive literature search for related studies are presented in Figure 1. A total of 54 potentially relevant articles were identified: PubMed (*n* = 5), ProQuest (*n* = 28) and ScienceDirect (*n* = 21), of which 50 articles were omitted after screening using inclusion and exclusion criteria. The following articles were excluded: 1 article examining bovine, 42 articles that were obviously irrelevant, 4 articles that were systematic reviews and 1 article that was focused on polycystic ovarian syndrome. On the basis of our research strategy, a total of four studies that met the inclusion criteria were identified, which included two papers using the Chinese population and two papers using the Japanese population. We only found two papers to be analysed for meta-analysis because the studies show the same SNP data, which is rs2746342 (Figure 1).

A total of four publications met the inclusion criteria, including 1,718 cases (T2DM) and 1,468 controls (healthy). Those references conducted studies in China and Japan. Three studies mostly involved male participants. Mean age with standard deviation of the case population ranged from 56.84 (SD 13.79) to 63.5 (SD 9.9), whereas for the control population, it was from 37.3 (SD 11.7) to 69.1 (SD 0.5). BMI among the three studies was the same, but one study did not describe BMI. The largest sample size (*n* = 1,760) was in the study conducted by Keshavarz et al. (26) and the lowest (*n* = 332) was in the study by Shen et al. (16). Based on subjects’ characteristics, the two studies included in the meta-analysis showed similarities in gender, age and BMI. Most patients were males, middle aged and overweight, except in the control group in Li et al. (17) that had normal-weight status.

The overall characteristics of the subjects are listed in Table 1. Cardiometabolic profiles, including FPG, TC, triglycerides, HDL-c and LDL-c (Table 2), were described in this review because they are closely related to T2DM risk. Table 2 shows that FPG, TC, triglycerides and LDL were nearly the same between those two studies included in meta-analysis. The mean of HDL-c in the case group in the study of Li et al. (17) was three times higher than that in the study of Shen et al. (16), which might contribute to the different results of those studies.

Table 3 describes the SNP type, detection method, genotype frequency, effect to T2DM and HWE in the four related articles. Methods to analyse genetic variation in the included studies were direct sequencing and Taq-man SNP genotyping assay. HWE analysis showed that all SNP *PRKAA2* in the four references are constant (*P* > 0.05). The four studies have observed 16 SNPs in the *PRKAA2* and showed a diverse result related to the risk of T2DM. Nevertheless, only five SNPs showed a significant correlation with T2DM risk, which were rs10789038, rs2796498 (17), rs2746342 (16), rs1418442 and rs932447 (26). Two included references among Chinese subjects detected similarity in SNP especially rs2746342. Li et al. (17) showed that rs2746342 has an insignificant association with T2DM risk, whereas Shen et al. (16) showed that subjects with genetic variation of rs2746342 have almost two times higher risk for significantly increased susceptibility for T2DM. Although rs2746342 in the study of Li et al. still contributed to T2DM after adjusting for other SNPs, Li et al. (17) and Shen et al. (16) described different results. In the future research, those two studies that detected rs2746342 need further investigation via meta-analysis to confirm their findings.

### Meta-Analysis Results

The effect model was applied appropriately to calculate the combined effect of two studies based on heterogeneity, which used *P*-value and I^2^ (%). Our results found that there was homogeneity between the two included studies in the meta-analysis, even in the subgroup analysis (*P* > 0.05 and I^2^ < 50%), so then we used a fixed-effect model for all genetic models. This finding might be caused by the same ethnicity, age, BMI and cardiometabolic characteristics, which were relatively the same between those studies.

**Figure 2:**
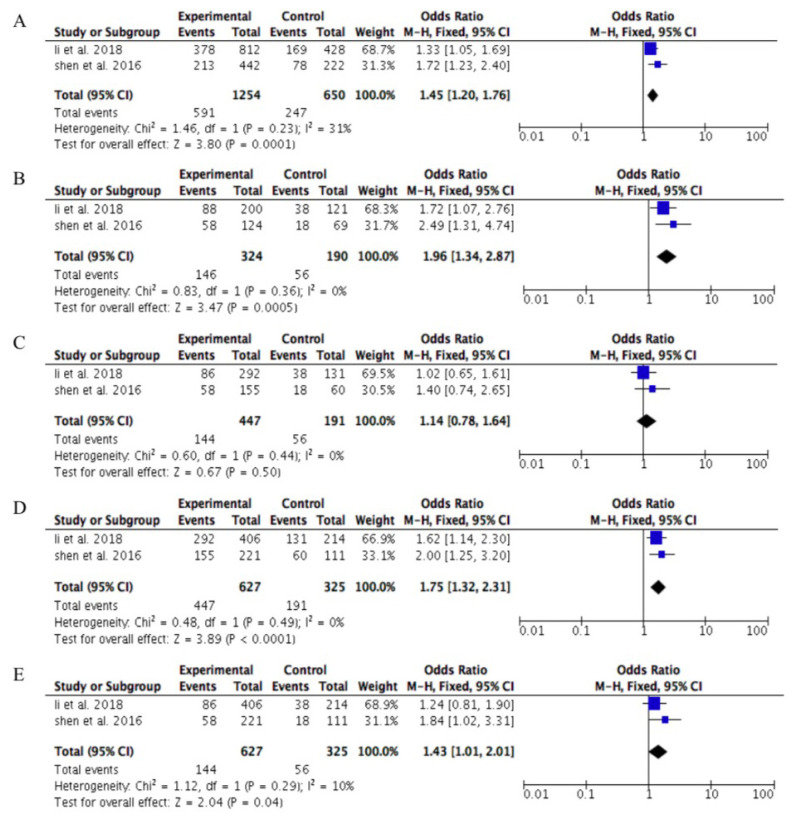
Forest plot of *PRKKA2* rs2746342 genetic variation in different models on the association with T2DM within studies conducted in Asia. **A**. Allele contrast model (G versus T allele); **B**. Additive model (GG versus TT); **C**. Additive model (GG versus TG); **D**. Dominant model (TG + GG versus TT) and **E**. Recessive model (GG versus TT + TG)

Although environmental factors have a contribution to T2DM, interestingly, our results showed that genetic factors, especially genetic variation in SNP rs2746342, had a significant correlation with T2DM risk. Meta-analysis results revealed that patients with G allele were 1.45 times more likely to develop T2DM compared with patients with T allele (OR: 1.45; 95% CI: 1.20, 1.76; *P*: 0.001). Additive model GG versus TT showed GG was 1.96 times higher in susceptibility to T2DM than TT (OR: 1.96; 95% CI: 1.34, 2.87; *P*: 0.0005). Dominant and recessive models showed a significantly different effect on the T2DM risk (TG+GG versus TT = OR: 1.75; 95% CI: 1.32, 2.31; *P*: < 0.0001 and GG versus TT+TG = OR: 1.43; 95% CI: 1.01, 2.01; *P*: 0.04). There was no significant difference in the GG versus TG additive model (OR: 1.14; 95% CI: 0.78, 1.64; *P*: 0.05) (Figure 2 and Table 4).

We conducted a subgroup analysis based on gender (Table 5). For the male group, there was an association that rs2746342 significantly increases the risk of T2DM in G versus T (OR: 1.48; 95% CI: 1.16, 1.89; *P*: 0.002), GG versus TT (OR: 1.99; 95% CI: 1.22, 3.24; *P*: 0.006) and TG+GG versus TT (OR: 1.56; 95% CI: 1.18, 2.06; *P*: 0.002). In contrast, the female group showed significant differences in only one genetic model, specifically the allele contrast model G versus T (OR: 1.43; 95% CI: 1.04, 1.95; *P*: 0.03). Notably, there were no significant differences between the male and female groups (*P* > 0.05).

## Discussion

Our review investigated the effect of *PRKAA2* variation on T2DM risk among Asians, specifically Han Chinese. To our knowledge, this is the first systematic review and meta-analysis that assessed the association between *PRKAA2* variation and the susceptibility of T2DM. AMPKα2, which is encoded by *PRKAA2*, is one of the AMPK units that is known to have a contribution in T2DM, especially in skeletal muscles (27). AMPK is an enzyme (serine/threonine kinase) that has an important role in lipid and glucose metabolism regulation. It also organises cellular energy homeostasis by changes to the catabolism pathway. AMPK is activated when cellular energy is low, and those signals will stimulate glucose uptake from skeletal muscles, reduce hepatic glucose production in the liver, and increase fatty acid oxidation in adipose. AMPK is located in the muscles, liver, heart, adipose tissue, pancreas and hypothalamus (23, 28). Thr-172 phosphorylation is needed in AMPK activation, and it is located in subunit α1 and α2 (21–22). However, the α2 subunit has a greater impact on lowering glucose and insulin resistance than the α1 subunit (24). Accordingly, AMPKα2 could be considered in T2DM predisposition factors. AMPK is also the main target in the mechanism of action in metformin (20–21), and to observe a relationship between *PRKAA2* variation and metformin effectiveness is a promising point of this research.

These findings showed that only 5 of 16 SNPs have a significant correlation with T2DM. First, rs10789038 could increase the risk of T2DM by 1.63 times in the AA genotype than in the GG genotype. Second, rs2796498 reduces T2DM risk in the GG genotype (17). Third, in rs2746342, the TT genotype had 0.75 times higher risk of T2DM than GG genotype (16). Fourth, the AA genotype in rs148442 had 0.62 times lower T2DM risk than the GG genotype. Last, in rs932447, the AA genotype had 0.62 times lower T2DM risk than the GG genotype (26). Those SNPs are located in PRKAA2, either in the intron or in the upstream transcript region. However, the association between SNP and the possible genetic predisposition for T2DM needs further investigation.

Nonetheless, only two SNPs, rs10789038 (17) and rs2746342 (16), indicated a higher risk of T2DM. SNP rs2746342 was the only SNP observed by two references that were done among Chinese. Interestingly, they exhibited different results, where one showed a significant association, but the other showed no significance. However, Li et al. (17) confirmed that rs2746342 has a strong correlation with the susceptibility of T2DM in haplotype analysis together with rs10780938 and rs2796498.

This meta-analysis hypothesised that *PRKAA2* variation affects T2DM risk, especially in SNP rs2746342. T allele as a mutant will increase the susceptibility of T2DM (29). In contrast, we found different results that the G allele (wild type) has a higher risk of T2DM than the T allele (mutant). Natural genetic predisposition in the Chinese population might have a higher risk of T2DM, so the G allele as wild type showed a higher risk than the mutant allele. China has the largest number of patients with T2DM (30) and the prevalence of T2DM in the Chinese population has been dramatically rising from 1980 to 2015 (31). Shen et al. (32) declared that the Chinese have a unique inheritable predisposition to T2DM risk compared with Caucasians. Indeed, it still needs further investigation because this review was coincidentally conducted in Chinese subjects.

It has been confirmed that the G allele has more potential for T2DM risk than the T allele. It could not present an association in GG versus TG model, but in GG versus TT showed the greatest association to T2DM risk. This result indicates that genetic variation rs2746342 could be a possible marker for early detection of T2DM risk. A high-risk population with a genetic variation in rs2746342 could in a preventive response change their environmental factors to minimise T2DM risk.

This review conducted a subgroup analysis by gender and using all genetic models. However, the GG versus TG relationship was found insignificant in overall results. Notably, there was no significant difference in the association between the male and female groups with T2DM based on *PRKAA2* rs2746342 genetic variation (*P* > 0.05). Nevertheless, this finding suggests that males tended to have a higher risk for T2DM than females, inclusively in SNP rs2746342. Genetic variation of rs2746342 in the male group had a significant relationship to increase the susceptibility of T2DM in almost all genetic models (*P* < 0.05), except in the additive model (GG versus TG and GG versus TT+TG). In contrast, the female group only had a significant association with T2DM risk in the allele contrast model G versus T (Table 5). It explains that the G allele is consistent in carrying T2DM risk, both in males and females. One epidemiology study described that T2DM risk is more significant in male than in female in the Asian population (33). Gender differences lead to differing sex-specific gene expressions, which influence the effect of sex hormones on organ systems (34).

However, not all studies found a correlation between *PRKAA2* and T2DM. A study of the Japanese population revealed no differences between wild-type and mutant alleles and genotype of *PRKAA2* in 10 SNPs for developing T2DM (35). SNP rs2746342 also had been studied as a risk factor of dyslipidemia in female Caucasians, which might contribute to increased cardiovascular risk. Nevertheless, these findings need further research in the Asian population.

There were some limitations that existed in our review. First, this study is not a representative population because we only included two studies in the meta-analysis, which involved only Han Chinese; one from Northern China, and the other from Southern China. Second, we could not perform further subgroups because of incomplete data and small sample sizes, particularly for the female groups.

## Conclusion

In summary, we could conclude that the PRKAA2 rs2746342 is related to increasing T2DM risk in the Asian population, especially in the Chinese, whether in G allele, additive, dominant and recessive models. The male group tended to have a greater risk for T2DM in correlation with rs2746342 genetic variation than the female group. G allele is the primary factor that increases the risk of T2DM. Accordingly, rs2746342 could be developed as a genetic marker of T2DM risk among the Chinese population. However, because of the limited area of the included studies, the conclusions of our review should be clarified by other studies that include larger, more diverse samples.

## Figures and Tables

**Table 1:** Characteristics of subjects from references included in the review

Reference	No. case/No. control	Ethnicity	Male/Female		Age mean (SD)		BMI mean (SD)	
		
			Case	Control	Case	Control	Case	Control
Li et al. (17)	406/214	Chinese	251/155	120/94	56.88 (12.60)	57.00 (7.98)	24.47 (2.26)	22.74 (1.71)
Shen et al. (16)	221/111	Chinese	153/68	76/35	56.84 (13.79)	51.14 (12.78)	25.74 (3.13)	24.25 (2.96)
Keshavarz et al. (26)	899/871	Japanese	448/451	428/443	63.5 (9.9)	37.3 (11.7)	NA	NA
Horikoshi et al. (35)	192/271	Japanese	123/69	129/143	61.3 (0.6)	69.1 (0.5)	24.1 (0.2)	23.9 (0.2)

Notes: BMI = body mass index; SD = standard deviation; NA = not available

**Table 2:** Cardiometabolic profiles related to T2DM from the studies included in the meta-analysis

Reference	Groups	FPG mean (SD)	Total cholesterol mean (SD)	Triglycerides mean (SD)	HDL-C mean (SD)	LDL-c mean (SD)
Li et al. (17)	Case	7.81 (2.51)	4.73 (1.28)	1.90 (1.87)	3.72 (50.28)	2.90 (3.16)
	Control	5.38 (1.51)	5.16 (4.31)	1.68 (3.7)	1.45 (0.49)	2.99 (4.75)
Shen et al. (16)	Case	7.74 (2.08)	4.84 (0.99)	2.31 (1.85)	1.10 (0.31)	2.66 (0.75)
	Control	5.15 (0.48)	4.94 (0.92)	1.77 (0.99)	1.17 (0.32)	2.73 (0.66)

**Table 3:** List of genotype studies included in the review

First author	Year of publication	Detection method	SNP	Genotype frequency			OR (95% CI)	HWE	
	
				Genotype	No. cases	No. control		*X* ^2^	*P*-value
Li et al.	2018	Sequencing	rs10789038	AA	292	135	1.63 (1.10, 2.43)	0.04	0.83
				GA	104	68			
				GG	10	11			
			rs2796498	GG	77	188	0.66 (0.45, 0.96)	0.92	0.34
				GA	97	180			
				AA	40	38			
			rs2746342	AA (TT)	114	83	0.75 (0.50, 1.12)	0.16	0.69
				CA (TG)	206	93			
				CC (GG)	86	38			

Shen et al.	2016	Sequencing	rs2746342	TT	66	51	1.99 (1.25, 3.20)	3.24	0.07
				TG	97	42			
				GG	58	18			
			rs2143754	AA	82	43	1.04 (0.64, 1.69)	0.51	0.47
				AG	101	50			
				GG	38	181			

Keshavarz et al.	2008	Taq-Man SNP genotyping assays	rs1418442	AA	544	517	0.62 (0.40, 0.96)	2.53	0.11
				AG	321	298			
				GG	34	52			
			rs932447	AA	539	518	0.62 (0.40, 0.96)	3.09	0.08
				AG	325	301			
				GG	34	52			

Horikoshi et al.	2006	Sequencing	−1439 A > T	AA	114	149	0.85 (0.62, 1.16)	1.02	0.31
				AT	71	110			
				TT	7	13			
			rs2051040	GG	64	106	1.21 (0.92, 1.58)	0.05	0.82
				GA	95	129			
				AA	33	37			
			rs2796492	CC	77	85	0.80 (0.61, 1.05)	0.18	0.67
				CT	87	143			
				TT	28	44			
			rs2796493	GG	76	83	0.78 (0.60, 1.04)	0.09	0.76
				GA	88	142			
				AA	28	47			
			rs2796495	GG	75	82	0.80 (0.60, 1.04)	0.01	0.91
				GA	90	146			
				AA	27	44			
			46991G > A	GG	134	176	0.81 (0.58, 1.14)	0.12	0.73
				GA	52	85			
				AA	6	11			
			rs2143754	TT	70	83	0.77 (0.59, 1.01)	0.00	0.98
				TC	92	128			
				CC	30	61			
			rs1418442	AA	110	144	0.89 (0.66, 1.21)	0.51	0.48
				AG	73	116			
				GG	9	12			
			rs932447	AA	113	155	1.04 (0.76, 1.42)	2.04	0.15
				AG	73	110			
				GG	6	7			
			rs3738568	TT	153	198	0.73 (0.49, 1.08)	1.34	0.25
				TC	35	68			
				CC	4	6			

Notes: SNP = single nucleotide polymorphism; OR = odds ratio; HWE = Hardy-Weinberg Equilibrium

**Table 4:** Meta-analysis results of rs2746342 for *PRKAA2*

Genetic models	Sample size		Association test			Heterogeneity test^a^		Model
		
	Case	Control	OR (95% CI)	Z	*P-*value	*P*-value	I^2^ (%)	
G versus T	1254	650	1.45 (1.20, 1.76)	3.86	0.0001	0.23	31	F
GG versus TT	324	190	1.96 (1.34, 2.87)	3.47	0.0005	0.36	0	F
GG versus TG	447	191	1.14 (0.78, 1.64)	0.67	0.50	0.44	0	F
TG+GG versus TT	627	325	1.75 (1.32, 2.31)	3.89	< 0.0001	0.49	0	F
GG versus TT+TG	627	325	1.43 (1.01, 2.01)	2.04	0.04	0.29	10	F

Notes: OR: odds ratio, F: fixed model;

a Heterogeneity test were used to determine fixed or random model would be applied

**Table 5:** Subgroup analysis of *PRKAA2* rs2746342 by gender (using fixed model effect)

Model	Group	OR (95% CI)	*P*-value	I^2^ (%)	*P*-value subgroup differences
G versus T	Overall	1.46 (1.20, 1.77)	0.0001	0	0.86
	Male	1.48 (1.16, 1.89)	0.002	0	
	Female	1.43 (1.04, 1.95)	0.03	23	
GG versus TT	Overall	1.89 (1.29, 2.76)	0.001	0	0.73
	Male	1.99 (1.22, 3.24)	0.006	0	
	Female	1.73 (0.94, 3.20)	0.08	0	
GG versus TG	Overall	1.14 (0.78, 1.65)	0.50	0	0.83
	Male	1.17 (0.73, 1.88)	0.51	0	
	Female	1.08 (0.60, 1.96)	0.71	0	
TG+GG versus TT	Overall	1.34 (1.09, 1.65)	0.005	6	0.11
	Male	1.56 (1.18, 2.06)	0.002	0	
	Female	1.11 (0.81, 1.52)	0.52	0	
GG versus TT+TG	Overall	1.37 (0.99, 1.89)	0.06	0	0.38
	Male	1.54 (1.01, 2.34)	0.05	0	
	Female	1.14 (0.68, 1.90)	0.62	0	

Note: OR = odds ratio
